# Early and Midterm Outcomes of Open and Endovascular Revascularization of Chronic Mesenteric Ischemia

**DOI:** 10.1007/s00268-020-05513-2

**Published:** 2020-04-23

**Authors:** Anna-Leonie Menges, Benedikt Reutersberg, Albert Busch, Michael Salvermoser, Marcus Feith, Matthias Trenner, Michael Kallmayer, Alexander Zimmermann, Hans-Henning Eckstein

**Affiliations:** 1 Department for Vascular and Endovascular Surgery and Munich Aortic Center (MAC), University Hospital Rechts der Isar, Technical University of Munich, Munich, Germany; 2 Department of Vascular Surgery, University Hospital Zurich, Zurich, Switzerland; 3 Department of Surgery, University Hospital Rechts der Isar, Technical University of Munich, Munich, Germany

## Abstract

**Background:**

Revascularization strategies for chronic mesenteric ischemia (CMI) include open (OR) and endovascular (ER) modalities. The primary objective of this study was to analyze the safety and effectiveness of OR and ER and the impact of clinical and morphological variables on early and midterm outcomes in a consecutive series of CMI patients in a tertiary referral center.

**Patients and methods:**

From 2004 to 2017, all CMI patients treated with OR and ER were retrospectively identified. Patient records, preoperative imaging, as well as peri- and postoperative outcomes were analyzed. Univariable and multivariable analysis was performed to identify clinical or morphological variables affecting reintervention rates within 2 years.

**Results:**

In total, 63 patients (33% male; mean age 71, range 60–76 years) were treated by ER (41 patients) or OR (22 patients) for CMI. Mean follow-up was 26 (10–71) months. 30-day mortality was 0.0% after ER and 4.5% after OR (*p *= 0.069); 30-day morbidity was 9.8% vs. 31.8%, respectively (*p *= 0.030). Length of stay was significantly longer after OR (14 vs. 4 days; *p *< 0.001). Freedom from reintervention rate after 2 years was 82% after OR and 73% after ER (*p *= 0.14). Overall survival did not differ after 2 years (OR 85% vs. ER 86%; *p *= 0.35). Multivariable analysis revealed that smoking was associated with higher risk of reintervention (hazard ratio, HR: 4.14; 95% confidence interval, CI 1.11–15.53; *p *= 0.03). Additionally, a nonsignificant trend of lower reintervention rates after OR was detected (HR 0.23 95% CI 0.05–1.08; *p *= 0.06).

**Conclusion:**

Due to a lower invasiveness, despite the higher reintervention rate, an “endovascular first” strategy is justified and recommended.

## Introduction

Although the prevalence of severe atherosclerosis of the mesenteric arteries supposedly ranges between 30 and 50% in the elderly population (> 65 years), the exact incidence of chronic mesenteric ischemia (CMI) is unknown. Clinically, CMI is a rarely diagnosed cause of abdominal pain [1, 2]. Scott J. Boley, a pioneer in the field of mesenteric ischemia, has been studying mesenteric circulation and vascular disorders of the intestines since the early 1960s. A particular concern was to focus physicians’ attention on the methods for, and potential success of, early diagnosis and aggressive treatment of these conditions [3, 4].

In approximately 90% of patients, it is caused by an atherosclerotic stenosis or occlusion of the superior mesenteric artery (SMA) and/or the celiac trunk (CTr) [5, 6]. Stenoses or occlusions of the inferior mesenteric artery (IMA) are usually clinically silent.

The most characteristic symptoms are postprandial abdominal pain, “food fear,” and subsequent unintended weight loss. However, patients can also present with nonspecific symptoms such as malaise, appetite loss, nausea, diarrhea, and weight loss of unclear origin [7]. The correct diagnosis is often made only after several months and, consequently, the majority of cases are recognized in late stages [8]. Persistent and refractory complaints are an indicator of advanced stage with chronic mesenteric malperfusion. Due to a vast collateral network of mesenteric vessels, symptoms usually only occur if more than one artery is affected. This is in contrast to acute mesenteric ischemia (AMI), where embolic occlusion of the SMA usually constitutes a life-threatening situation [9].

Revascularization has been proven to be beneficial in cases of symptomatic CMI, regardless of the number of affected vessels [10]. Additionally, invasive treatment can be indicated in selected cases of non-symptomatic atherosclerotic mesenteric arteries, to prevent AMI in the further course [11].

While computed tomography angiography (CTA) is the diagnostic method of choice, the optimal revascularization strategy for individual patients sometimes remains unclear [12].

The first successful open revascularization (OR) for CMI was reported in 1958 by RS Shaw from the Massachusetts General Hospital when performing endarterectomy of the SMA [13]. Due to the rapid development of endovascular techniques and the assumed lower procedural morbidity and mortality compared to OR [14–16], the therapeutic approach has progressively moved from open repair (OR) to a primary endovascular approach. ER is now applied initially in 70–80% cases [15, 17].

Since randomized controlled trials (RCTs) comparing OR and ER are lacking, the primary objective of this study was to examine early and midterm outcomes in a consecutive series of CMI patients treated by OR and ER at a tertiary referral center. In particular, clinical and morphological variables and their potential impact on reintervention rates and long-term survival were analyzed.

## Patients and methods

This study is a retrospective analysis of a consecutively treated cohort of CMI patients at a single university hospital center between January 2004 and December 2017. Identification of the study patients was based on the hospital information system that includes all medical records. Patients were retrieved using the German modification of the International Classification of Diseases (ICD)-10 code for CMI (K55.1). Inclusion criteria were surgical or endovascular treatment of CMI. Patients who required additional surgical treatment (e.g., for concurrent aortic aneurysm repair or renal revascularization) and patients with acute mesenteric ischemia (AMI) were excluded.

### Diagnostic and surgical strategies

All patients underwent CTA to assess individual anatomy and determine the feasibility of an endovascular approach. All CTAs were evaluated by an interdisciplinary vascular board (vascular surgery, radiology, and cardiology) for different therapeutic strategies. The choice of therapy was affected by anatomy, comorbidities, the urgency of repair, and the patient’s preference. ER was performed by percutaneous transluminal angioplasty (PTA) and/or stenting of one or more visceral vessels under local anesthesia whenever possible. OR was performed by transposition of the SMA (or IMA in one patient) into the aorta, bypass grafting, or endarterectomy.

### Data acquisition

Baseline clinical data included sex, age, clinical presentation (postprandial abdominal pain, weight loss, diarrhea, and duration of abdominal complaints), surgical and medical history, and comorbidities. Specific data included the number of affected mesenteric arteries, surgical strategy, primary technical success, length of stay (LoS), complications (cardiac, respiratory, renal, gastrointestinal, wound healing, and puncture site complications), and reintervention rates. Morphological data included type, length, location of lesion, previous interventions, and grade of calcification as described by Zacharias et al. [18]. For calcification scoring, two experienced readers separately evaluated the CTA data. Disagreements were solved by consensus. The circumference of the affected vessel was divided into thirds and classified into low-, middle-, and high-grade calcification, similar to methods reliably used in the coronary or carotid arteries [19]. Technical success was defined as residual stenosis of less than 30% by angiography.

Data acquisition was in accordance with the Declaration of Helsinki and written informed consent was obtained from all patients.

Follow-up was carried out in accordance with a standardized protocol and included a clinical examination and duplex ultrasound of the visceral arteries 6 weeks after discharge and annually thereafter in an outpatient setting. Patients with recurrent symptoms were additionally evaluated with CTA or diagnostic angiography. If not seen within the standard surveillance protocol, patients or their family doctors were contacted by phone and an appointment was made for the patient for a follow-up evaluation at the time of data collection in March 2018.

### Endpoints

Primary endpoints were 30-day mortality (safety endpoint) and freedom from reintervention (efficacy endpoint). Secondary endpoints were perioperative (30-day) morbidity, LoS, and overall survival (OS).

Technical success in the case of ER was defined as residual stenosis of less than 30%, confirmed at the end of the endovascular procedure. In OR cases, technical success was characterized by patent reconstruction of the visceral arteries. Clinical success was defined as resolution of clinical symptoms within 3 days after OR or ER. Medical complications within the first 30 days postoperatively defined 30-day morbidity, whereby we differentiated between seven groups of complications.

### Statistics

Categorical data were compared using Fisher’s exact and Chi-square tests, and continuous data were assessed by the Mann–Whitney U test. Primary patency and survival rates were estimated using Kaplan–Meier analysis and compared with a log-rank test. Univariable and multivariable analysis on demographic, clinical, and morphological characteristics was performed to assess their association with the risk of reintervention. For the primary safety and efficacy endpoints, a baseline Cox proportional hazards model was fitted using demographic variables (age and sex) only. Subsequently, another clinical or morphological variable was added to the baseline model, evaluated, and finally removed again. Statistical analyses were performed using Med-Calc© version 9.6.4.0 (MedCalc Software, Mariakerke, Belgium), with *p *≤ 0.05 considered statistically significant.

## Results

### Patient characteristics (Table 1)

A total of 63 patients (33% male; mean age 71, range 60–76 years) fulfilled the inclusion criteria. Over the study period, an increasing number of patients (2002 *n *= 2, 2017 *n *= 8) were treated with ER.

**Table 1:** Patient demographics, comorbidities, and morphological criteria

	Total (*n *= 63)	ER (*n *= 41)	OR (*n *= 22)^a^	*p* value
*Demographics*				
Age (years) median (range)	71 (60–76)	71 (64–78)	70 (53–73)	0.145
Sex (male%)	21 (33.3)	11 (26.8)	10 (45.5)	0.137
*Symptoms*				
Weight loss	38 (60.3)	22 (53.7)	16 (72.7)	0.145
Postprandial pain	53 (84.1)	36 (87.8)	17(77.3)	0.281
Nausea and vomiting	12 (19.0)	7 (17.1)	5 (22.7)	0.593
Diarrhea	15 (23.8)	12 (29.3)	3 (13.6)	0.167
Symptom onset (months), median (range)	3 (0.5–11)	5 (0.5–14)	2 (0.6–6)	0.165
*Comorbidities*				
Hypertension	40 (63.5)	27 (65.9)	13 (59.1)	0.596
Hyperlipidemia	25 (39.7)	17(41.5)	8(36.4)	0.696
CHD	25 (39.7)	18 (43.9)	7 (31.8)	0.353
Ever smoker	31 (49.2)	21 (51.2)	10 (45.5)	0.669
Diabetes	20 (31.7)	13 (31.7)	7 (31.8)	0.994
ESRD	7 (11.1)	4 (9.8)	3 (13.6)	0.650
*Morphological parameter*				
High-grade calcification	36 (57.1)	23 (56.1)	13 (59.1)	0.820
Middle-grade calcification	8 (12.7)	5 (12.2)	3 (13.6)	0.875
Low-grade calcification	18 (28.6)	13 (31.7)	5 (22.7)	0.455
Location of vessel occlusion > 2 cm from origin	15 (23.8)	9 (22.0)	6 (27.3)	0.641
Length of stenosis > 2 cm	9 (14.3)	4 (9.8)	5 (22.7)	0.167
Occlusion	11 (17.5)	4 (9.8)	7 (31.8)	**0.030**
Previous interventions	8 (12.7)	6 (14.6)	2 (9.1)	0.535

When not stated otherwise, results are given as numbers (%)

*ER* endovascular repair, *OR* open repair, *CHD* coronary heart disease, *ESRD* end-stage renal disease

^a^2 patients were converted after failed ER within 7 days and further considered as OR

*p* values were calculated by Mann–Whitney *U* or Chi-square tests, significant difference is highlighted in bold

Main clinical symptoms were abdominal angina and weight loss, found in 84 and 60%, of patients, respectively. Atherosclerotic risk factors, comorbidities, and patient demographics were quite evenly distributed between the OR and ER groups. Most patients (87%) had no prior surgical or endovascular intervention of the mesenteric arteries. However, the majority (80%) had already been treated for atherosclerotic diseases in another vascular region.

### Morphological variables (Table 1)

Of all patients, 60% suffered from multivessel disease. In 89% of patients, the SMA was affected. Whereas the calcification grade did not differ between OR and ER, significantly more occlusions were treated in the OR group (32 vs. 10%, *p *= 0.030).

### Surgical treatment (Table 2)

ER was used in 41 patients (45 arteries) and OR in 22 patients (24 arteries) and was performed by angioplasty alone in 17/41 patients (17 arteries). An additional stent was used in 24/41 patients (24 arteries). The most common procedure in the OR group was transposition of the SMA in the aorta or aortomesenteric bypass surgery (14/22 patients). In both groups, usually only one visceral artery was treated (OR 80% vs. ER 86%). The technical success rate was 88% in the ER group and 100% in the OR group. Two patients had to be converted to OR after failed ER within 7 days. Further, they were considered to OR group.

**Table 2:** Technical details and comparison of 30-day peri- and postoperative outcomes for open (OR) and endovascular repair (ER) of chronic mesenteric ischemia

	Total (*n *= 63)	ER (*n *= 41)	OR (*n *= 22)	*p* value
*Technical procedures*				
Number of artery (*n*)	69	45	24	N/A
Angioplasty	–	17 (18)	–	N/A
Stenting	–	24 (27)	–	N/A
Failure of endovascular therapy	–	2 (2)	–	N/A
Endarterectomy	–	–	5 (7)	N/A
Mesenteric bypass or transposition	–	–	14 (14)	N/A
Thrombectomy	–	–	1 (1)	N/A
Resection of arcuate ligament	–	–	2 (2)	N/A
*Early outcome*				
30-day morbidity	11 (17.5)	4 (9.8)	7 (31.8)	**0.030**
30-day freedom from reintervention	96.7% (92.4–100)	97.4% (92.6–100)	95.5% (87.1–100)	0.069
30-day mortality	1.6% (0 – 4.8)	0% (0–0)	4.5% (0–2.9)	0.768
Length of stay (days)	7 (3–12.5)	4 (3–7)	14 (10–17)	**< 0.001**

Numbers represent median and range (Q1–Q3); technical details are given in number of patients *n* (number of arteries n); *p* values were calculated by Mann–Whitney U or Chi-square test, significant differences are highlighted in bold

### Early outcomes (Table 2)

Mortality (30-day and in-hospital) was 1.6% (0% for ER; 4.5% for OR; *p *= 0.069), with one fatality on day 12 due to necrotizing pancreatitis. A total of 11 patients (17.5%) suffered from perioperative complications (30-day morbidity), with significantly higher numbers in the OR group as compared to ER (31.8% vs. 9.8%; *p *= 0.030).

There were mild complications in the ER group in three cases (angina pectoris (*n* = 1), ischemic rectitis (*n* = 1) and one false aneurysm in the femoral puncture site (*n* = 1)) and severe complications in one case (pneumonia and urosepsis). In the OR group, five of seven patients had severe complications (myocardial infarction (*n* = 2), pneumonia (*n* = 1), acute renal failure (*n* = 1), pancreatic fistula (*n* = 1), and pancreatitis (*n* = 1)) and two mild complications (postoperative delirium, deep vein thrombosis).

LoS was longer after OR compared to ER (14, Q1–Q3: 10–17 vs. 4:Q1–Q33–7 days, *p *< 0.001).

The freedom from reintervention rates at 30 days was 96% vs. 97% after OR and ER, respectively (*p *= 0.069).

### Midterm and late outcomes (Tables 2, 3)

Clinical success was achieved in 95% in both groups. The remaining 5% had persistent complaints despite technical success; initial misdiagnosis thus had to be considered, and patients were transferred to the gastroenterology department.

**Table 3:** Univariable analysis of clinical, morphological, and operative variables and their impact on the 2-year rates of reintervention after endovascular or open revascularization for chronic mesenteric ischemia

	HR(95% CI)	*p* value
*Baseline characteristics*		
Age ≥65 versus <65 years	0.64 (0.21–1.90)	0.42
Female/male	1.50 (0.49–4.60)	0.48
*Clinical parameter*		
Hypertension (yes/no)	1.67 (0.51–5.44)	0.39
Hyperlipidemia (yes/no)	1.32 (0.44–3.93)	0.62
CHD (yes/no)	1.87 (0.63–5.57)	0.26
Ever smoker (yes/no)	3.91 (1.08–14.23)	0.04
Diabetes (yes/no)	2.46 (0.82–7.35)	0.11
ESRD (yes/no)	1.52 (0.34–6.88)	0.58
*Morphological parameter*		
High-grade versus low-/mid-grade calcification	1.15 (0.39–3.45)	0.80
Stenosis/occlusion >2 cm versus <2 cm from origin	1.07 (0.29–3.90)	0.92
Lesion length >2 cm/<2 cm	1.67 (0.46–6.10)	0.44
Occlusion versus stenosis	1.16 (0.32–4.23)	0.82
Previous interventions (yes/no)	1.84 (0.51–6.68)	0.36
*Mode of revascularization*		
OR versus ER	0.27 (0.06–1.23)	0.09

*HR* hazard ratio, *CI* confidence interval, *OR* open revascularization, *ER* endovascular revascularization, *CHD* coronary heart disease, *ESRD* end-stage renal disease

*P* values were calculated by Mann–Whitney *U* or Chi-square tests, significant differences are highlighted

Mean follow-up was 26 (Q1–Q3: 10–71) months. Longer follow-up was available in 18 patients (28.5%). In Kaplan–Meier analysis freedom from reintervention rates within 2 years were 82% for OR and 73% for ER (*p* = 0.14). Recurrent stenosis with or without symptoms indicated reintervention.

There was also no significant difference between OR and ER in Kaplan–Meier analysis for OS, with 30-day and 2-year survival rates of 95 and 85% for OR, and 100 and 86% for ER, respectively (*p *= 0.35).

**Fig. 1:**
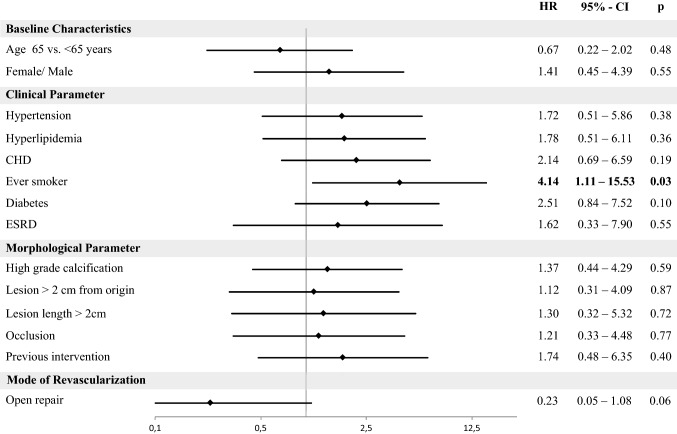
Clinical, morphological parameters and mode of revascularization were adjusted for age and sex. HR, hazard ratio; CI, confidence interval; OR, open revascularization; ER, endovascular revascularization, CHD, coronary heart disease, ESRD, end-stage renal disease

In the uni- and multivariable analysis (Table 3, Fig. 1) adjusted for clinical and morphological parameters, a positive smoking history or current smoking was associated with a significantly higher risk of reintervention (hazard ratio, HR: 4.14; 95% confidence interval, CI 1.11–15.53; *p *= 0.03; Fig. 1), while OR tends toward a lower reintervention risk (HR 0.23, 95% CI 0.05–1.08; *p *= 0.06).

## Discussion

In this consecutive cohort of patients suffering from chronic mesenteric ischemia (CMI), there were no significant differences in terms of reintervention rate, 30-day mortality, or overall survival (OS) between endovascular (ER) or open vascular surgical revascularization (OR) during a mean follow-up of 26 months. As with many other studies in the field, most likely due to the small sample number, we only observed trends for a lower reintervention rate in OR and better OS in ER. OR was associated with a higher rate of 30-day morbidity and longer LoS. In multivariable analysis, smoking history was associated with higher rates of reintervention.

As in other studies, a significant higher perioperative morbidity for OR in our cohort was observed, while there was no significant difference between ER and OR in terms of 30-day mortality (Table 2) [20]. Overall, 30-day mortality in the OR group was 4.5% and thus almost identical to the 5.5% of a recently published meta-analysis [21]. However, perioperative mortality due to cardiovascular events can be as high as 15% after OR [22]. In our department, a complete preoperative cardiologic workup including possible noninvasive imaging in all elective patients precedes treatment.

However, due to the lower invasiveness of ER, there are not only differences in perioperative morbidity, but also in LoS. These results have been confirmed in various other studies [20, 22, 23]. In addition, lower costs could be calculated for ER compared to OR [24, 25].

Attention should be paid to the fact that not all lesions in our cohort are suitable for ER, because of long-distance occlusions. In our study, seven patients who were primarily treated with OR would not have been eligible for an endovascular procedure, which may well have led to a bias in favor of ER.

One of the main problems of endovascular treatment in general is its durability, mostly in terms of target vessel patency [26]. Some of the newer methods (e.g., drug eluting balloons or stents) are not yet used in clinical routine for treating mesenteric vessels. However, although freedom from reintervention showed no significant difference between ER and OR in our study, 30-day and 2-year freedom from reintervention showed a tendency toward favoring OR (albeit without statistical correction for various endovascular means applied). This is likely due to the low case number, and those patients lost to follow-up. Another reason for the high reintervention rate could be the treatment without stenting. In our study, nearly half of the ER group (17 of 41 patients) was treated with angioplasty alone. Prospective trials to compare angioplasty with primary stenting are missing. But experts agree, and the 2017 Guidelines from the European Society for Vascular Surgery (ESVS) recommend that primary mesenteric stenting is indicated [9, 27–30].

Nevertheless, the reintervention rate of 27% by ER after 2 years is in accordance with the published results [20, 31]. Therefore, reintervention rates and worse primary patency rates need to be considered when opting for an “endo-first” strategy [22, 32].

Moreover, the multivariable analysis of the current study confirmed a tendency of lower reintervention rates for OR. Thus, OR remains a viable option in patients who are fit for surgery and are morphologically no good candidates for ER. Particularly, an early change of regimen after failed ER should be considered as supported by the ESVS guidelines [9].

Decision-making should also support clinical or morphological variables which might be associated with lower patency rates [33]. Even though the numbers of CMI patients were relatively low in this study, our multivariable analysis revealed that patients with a history of smoking or current smoking have a fourfold higher risk of reintervention. This seems plausible, since although hit has not been described for mesenteric arteries, other investigations of different areas and techniques have reported similar results regarding smoking as a risk factor for restenosis [34–38]. Regarding the complexity of lesions, Oderich et al. investigated endovascular procedures. In this study, female sex as well as long (< 20 mm) and calcified lesions significantly increased the risk of in-stent restenosis [31, 39]. These factors were also analyzed in our cohort, but did not show the significant results due to the small sample size (Table 3).

Not only the high rate of reintervention plays an important role in arteriosclerotic disease, but also the poor overall survival has (OS) [40]. Patients with severe atherosclerotic burden have a poor survival prognosis; for example, patients with chronic limb threatening ischemia have a 1-year survival probability of only about 75% if affected by CMI [41]. However, even patients with CMI alone have a poor OS [42]. Therefore, it is satisfying that OS in this study was 98.4% at 30 days and 86.1% at 2 years. We were not able to detect any difference between the two treatment options, and these results thus correspond to the results of the 2018 published meta-analysis, where also no significant difference in the 3-year OS of 78% was found.

Another treatment option that should be mentioned but was not used in our cohort is a hybrid procedure with retrograde stenting of SMA. This option may be selected when transaortic stenting and open reconstruction are impossible, e.g., in case of extensive aortoiliac disease and no good source of inflow, and it is mainly recommended in those with acute mesenteric ischemia. The use of a hybrid approach provides one of the most expeditious methods of revascularization in patients with difficult SMA occlusions [9, 43–45].

Several shortcomings of this study need to be addressed. First, selection bias is possible as it is a single-center analysis. Second, the cardiovascular and morphological risk factors used in the multivariate analysis represent only a small number of the factors influencing OS and freedom from reintervention. Finally, this study had the inherent limitations of a retrospective analysis.

## Conclusion

CMI is a rare disease that can be effectively treated by ER and OR. ER tends to have higher rates of reintervention, while 30-day morbidity is higher and hospital LoS is longer in patients treated with OR. Due to the less invasive nature of ER, the results support the “endovascular-first” strategy that has been established in recent years.
